# The Foreign Journals: Toxicology

**Published:** 1839-04

**Authors:** 


### TOXICOLOGY.

### *Case of Poisoning by Arsenious Acid successfully treated by the Hydrated Tritoxide of Iron.* By Dr. DEVILLE.

A young lady, in consequence of a disappointment in a love affair, determined to poison herself, and accordingly one night, a little before twelve o'clock, took (mixed in water) a considerable quantity of arsenic powder, which had been bought to destroy rats. The symptoms of poisoning commenced about one o'clock in the morning with vomiting, and she brought up part of the powder with some undigested food. Dr. Deville did not see her before four o'clock, A.M., when she had vomited several times, was suffering dreadful pain in the head and stomach, with burning heat in the throat, and seemed in a very dangerous state. She was immediately given a quantity of milk and linseed decoction, to encourage the vomiting and to assist the discharge of the poison; but this and other means failed to give any relief, and the symptoms became more urgent. Dr. Deville then determined to give hydrated tritoxide of iron; but as he had some difficulty in procuring this, it was half-past five before he returned to the patient. He immediately administered about half an ounce of the antidote by the mouth, and repeated this dose every quarter of an hour, so that by eight o'clock, A.M., she had taken more than half a pound of the iron. He then stopped, as the medicine had produced vomiting and purging, and the urgent symptoms seemed somewhat abated; twenty-five leeches were then applied on the epigastrium, with other general treatment, and the patient gradually recovered. In twelve days she was perfectly well. From a careful examination of the poison which remained in the glass, out of which she had taken it, and from knowing the quantity of the powder originally contained in the packet, Dr. Deville came to the conclusion that

56 grains of arsenious acid had been taken into the stomach, and remained there for above an hour; and though a considerable part (which was thrown away) might have been discharged by vomiting, still a large quantity, he conceives, remained in the stomach.

[The preservation of the patient's life, in the opinion of Dr. Deville, was incontestibly owing to the hydrated tritoxide of iron; but the case is open to considerable objections, for the greater part or the whole of the poison might have been discharged with the vomited matters, which unfortunately were not preserved. Six cases of poisoning by arsenic in the human subject, successfully treated by tritoxide of iron, by Messrs. Geoffrey, Biteau, and Majesté, are related in Vol. I. p. 572-3 of this Journal, where the mode of preparing the antidote is also mentioned. In Vol. I. pp. 594-5, and in Vol. IV. p. 237, are related several experiments on animals with the hydrated peroxide of iron, as an antidote to arsenious acid.] ***Rev. Méd. Fran. et Etran.*** Sept. 1838.

---

*Toxicological Experiments, with Preparations of Chromium.* By Dr. BERNDT.

I. *Experiments with bichromate of potash.* EXP. I. Fifteen grains of the salt, dissolved in 2 ounces of water, were given to a full-grown rabbit. Five minutes afterwards the animal took food; in twenty minutes the respiration became irregular, and thirst supervened. It now received a second dose of 15 grains, which produced very anxious respiration, and attempts, as it were, at vomiting, which were rendered abortive by the peculiar structure of its stomach. In about an hour and a quarter, the fore-feet were seized with trembling; in two hours the strength had sunk very much, and there was also incipient paralysis of the hind-feet. Death ensued in two hours and twenty minutes, with convulsions and evacuation of the fæces and urine. The body was examined two hours after death; the jaws were firmly closed; the cerebrum, cerebellum, and spinal marrow were gorged with blood, and somewhat softer than usual; the trachea and lungs were likewise gorged, the heart was flabby, and the right auricle was filled with fluid blood. The stomach was injected, its mucous membrane was partially destroyed, and its contents, when tested, showed the presence of chromium. The intestines were filled with mucus. The liver appeared softened.

EXP. II. A pigeon received 30 grains, made into a pill, with crumbs of bread, in doses of 10 grains, with an interval of half an hour between each dose. Soon after the last dose the animal became rigid, and made ineffectual attempts to vomit. It died in about four hours with slight convulsions. Its body was examined three hours after death; the body was rigid, the brain gorged with blood in a remarkable degree, and it, as well as the spinal cord, appeared softer than natural. The crop was injected, and contained nearly the whole of the pills as they had been swallowed; the stomach contained some chromium; the whole intestinal canal was reddened; the heart was flabby; and the lungs were gorged with blood.

EXP. III. A wound was made in the neck of a moderate-sized bitch, and 30 grains of the salt were introduced beneath the integument, and the wound was then stitched up. Frequent evacuation of the bowels and bladder followed in about a quarter of an hour, and immediately afterwards vomiting and trembling. At first the matter vomited consisted merely of the food; it then became mucous, and after some hours resembled the serum of blood. The usual reagents showed no indications of the presence of chromium. In eight hours the hind-feet were paralyzed, the fore-feet continued to tremble violently. The animal died in eleven hours. The body was examined ten hours after death; it was rigid, and had an unpleasant smell; the wound was dry, whitish, and contained a trace of the salt; the cerebrum and cerebellum were gorged with blood, and, along with the medulla, much softened. The lungs were filled with blood, as was also the right side of the heart. The stomach and upper portion of the intestinal canal, and the bladder, were reddened, but their contents showed no trace of the presence of chromium.

[Sufficient care does not appear to have been here taken in testing for chromium.

The metallic salts, it is known, are capable of forming definite compounds with animal and vegetable substances; and in such cases their presence cannot be detected by the usual reagents. It is necessary to destroy all traces of organic matter before any reliance can be placed upon the indications afforded by the reagents.]

EXP. IV. A dog, three months old, received 4 grains in half an ounce of water. In about half an hour vomiting ensued, and most of the solution was evacuated. The vomiting then ceased; the dog remained for some time very weak, but eventually recovered.

EXP. V. Three grains in powder were given to a frog. (In the experiments with frogs they were placed in water, but not deep enough to cover the head.) Vomiting ensued immediately, and part of the undissolved salt was evacuated; the vomiting, however, continued, and the animal died with convulsions in about an hour. The body was examined immediately; there was no rigidity; the brain and spinal cord were softened; the lungs were dilated and traversed by red vessels; the heart was flabby. The intestinal canal was not much injected, and the contents of the stomach showed the presence of chromium.

EXP. VI. A frog received 2 grains in pills. The consequences were similar to those of EXP. V.

EXP. VII. Five grains were dissolved in 4 oz. of water, and a frog was placed in the solution. In a quarter of an hour it became restless for a short time; in about an hour the restlessness returned, and continued to increase till, in about two hours, it attained its maximum. In eight hours spasms of the hind-feet came on, with retching and vomiting. It died in about twelve hours, and the body was examined ten hours afterwards. It was very rigid; the heart was flabby, and like the lungs gorged with blood. The brain and spinal cord were not examined.

EXP. VIII. A frog was placed in a solution containing 20 grains to 4 oz. of water. The phenomena were similar to those observed in the preceding case, but more violent, and death ensued in three hours and a half. The brain and spinal cord were very soft; the lungs small and dark coloured, the heart filled with blood. The throat, stomach, and intestines were highly injected.

EXP. IX. A frog was placed in a solution containing 20 grains to 2 oz. of water. The results were similar to, but more violent than those of the two preceding cases.

EXP. X. A frog was kept for forty-five minutes in a solution containing 20 grains to 2 oz. of water. It was then taken out of the solution, washed, and placed in fresh water. The removal produced no change in the phenomena, and the animal died in four hours.

EXP. XI. A solution of 10 grains in 3 drachms of water were injected into the jugular vein of a dog four months old. The animal cried violently and died instantly. The body was immediately opened: all the blood in the cavity of the thorax was found coagulated; the heart was very much dilated, and its right half continued to pulsate after the left had ceased. The blood in the brain was partially, in the abdomen not at all, coagulated.

II. *Experiments with the chromate of potash.* These experiments were conducted in a similar way to those already related; and the effects of the chromate resemble very nearly those of the bichromate. Perhaps the latter salt is fully more powerful than the former; but the details of the experiments are so much like those we have already given, that we think it needless to enter more fully upon them.

III. *Experiments with the protoxide of chromium.* These experiments merely prove that the protoxide is an inert powder.

IV. *Experiments with substances which it was conceived might prove to be antidotes.* These experiments consisted in first poisoning the animals with the chromate or bichromate of potash, and then giving carbonate of potash, sulphate of iron, or tincture of galls, as antidotes. These substances, however, did not in any way neutralize the effects of the poison, and we need not, therefore, detail the experiments. *Medicinische Zeitung.* Nos. 24 and 25. 1838.

